# *EGFR* methylation and outcome of patients with advanced colorectal cancer treated with cetuximab

**DOI:** 10.3892/ol.2015.2876

**Published:** 2015-01-14

**Authors:** ELISA CHIADINI, EMANUELA SCARPI, ALESSANDRO PASSARDI, DANIELE CALISTRI, MARTINA VALGIUSTI, LUCA SARAGONI, WAINER ZOLI, DINO AMADORI, PAOLA ULIVI

**Affiliations:** 1 Biosciences Laboratory, Istituto Scientifico Romagnolo per lo Studio e la Cura dei Tumori (IRST) IRCCS, Meldola, Italy; 2 Unit of Biostatistics and Clinical Trials, Istituto Scientifico Romagnolo per lo Studio e la Cura dei Tumori (IRST) IRCCS, Meldola, Italy; 3 Department of Medical Oncology, Istituto Scientifico Romagnolo per lo Studio e la Cura dei Tumori (IRST) IRCCS, Meldola, Italy; 4 Pathology Unit, Morgagni-Pierantoni Hospital, Forlì, Italy

**Keywords:** methylation, colorectal cancer, epidermal growth factor receptor, cetuximab

## Abstract

Targeted therapy of metastatic colorectal cancer (mCRC) with monoclonal antibody anti-epidermal growth factor receptor (EGFR) agents, such as cetuximab (CTX) or panitumumab, is the treatment strategy of choice in patients characterised by a wild-type (wt) *RAS* gene status. However, despite selection based on *RAS* status, a high proportion of patients do not respond to therapy. *EGFR* methylation has been reported to have a role in predicting the response to anti-EGFR agents. The present study aimed to evaluate the role of *EGFR* methylation in association with the clinical outcome of patients with mCRC treated with CTX. In total, 64 patients with mCRC were assessed in the present study. Genomic DNA was extracted from tumoral tissue and *EGFR* methylation and mutation of the *KRAS*, *BRAF* and *PIK3CA* genes were analysed by pyrosequencing. EGFR expression was assessed by immunohistochemistry. The various alterations were analysed by assessing the objective response rate (ORR), progression free survival (PFS) and overall survival (OS) rates. In total, 42 cases (66%) exhibited >10% EGFR methylation and there was no correlation with EGFR expression. Mean *EGFR* methylation of 41 and 9% was observed in *KRAS*-mutated and -wt patients, respectively (P=0.05). Conversely, a high *EGFR* methylation was observed in *BRAF*-wt patients with compared with patients possessing the mutated gene (18 vs. 3%, respectively; P=0.07). *EGFR* methylation was significantly correlated with the OS rate [hazard ratio, 0.98; 95% confidence interval (CI), 0.96–1.00; P=0.019], but not PFS rate. In patients with a methylation rate <10 and >10%, the median OS rate was 7.5 months (95% CI, 4.4–9.4 months) and 12.0 months (95% CI, 8.7–13.9 months), respectively (P=0.034). In conclusion, the present study revealed a correlation between *EGFR* methylation and improved OS rate in patients treated with CTX-based chemotherapy. The presence of *EGFR* methylation is inversely correlated with *BRAF* and *PIK3CA* mutations, indicating that the prognostic value of gene methylation may be worth verifying in further studies.

## Introduction

Targeted therapy of metastatic colorectal cancer (mCRC) using monoclonal antibody (moAb) anti-epidermal growth factor receptors (EGFR) agents, such as cetuximab (CTX) or panitumumab, is the treatment strategy of choice in patients characterised by a wild type (wt) *KRAS* gene status (1–3). Despite the selection for *KRAS*-wt patients, only ~25% of patients demonstrate a response to treatment. Several studies have attempted to identify additional potential biomarkers for the selection of patients who are likely to respond to therapy, or to exclude those who are not likely to respond (4–12). *BRAF* and *PIK3CA* are the most frequently studied genes, and their mutations have been analysed in association with CTX response. In particular, a number of studies have demonstrated that *BRAF* and *PIK3CA* mutations were associated with a worse objective response rate (ORR), progression free survival (PFS) rate and overall survival (OS) rate, compared with wt patients, suggesting that the selection of triple-wt patients for *KRAS*, *BRAF* and *PIK3CA* mutations may be a good strategy for improving the outcome of patients treated with CTX or panitumumab (4,5,7–10,12). Although several studies have revealed a possible role of the two genes as predictive markers, others have highlighted their role as prognostic, rather than predictive, factors (13–16). *NRAS* mutations have also been added in clinical practice, together with *KRAS*, as a discriminating marker for moAb anti-EGFR drugs, as it has been demonstrated that patients with a mutated *NRAS* gene did not respond to therapy (17).

In addition, although the EGFR protein represents the target of therapy, protein expression does not appear to be a potential indicator of response (18).

A previous study has demonstrated that *EGFR* promoter methylation, an epigenetic event associated with the loss of EGFR expression, is associated with a poor prognosis for patients, highlighting the possibility that immunohistochemistry may not be the optimal method for EGFR expression assessment and that, EGFR expression may aid in predicting the effect of anti-EGFR therapies (19). However, the association between *EGFR* methylation and the other common alterations, including *KRAS*, *BRAF* and *PIK3CA* mutations, was not analysed.

The present study aimed to analyse the correlation between *EGFR* methylation and the clinical outcome of patients in a case series of mCRC patients treated with CTX-based chemotherapy. The association between EGFR methylation and *KRAS*, *BRAF* and *PIK3CA* mutations was also analysed.

## Patients and methods

### Patients

In total, 64 consecutively enrolled patients with mCRC treated with a CTX-based regimen at IRST IRCCS (Meldola, Italy) between March 2004 and October 2010 were retrospectively analysed for the present study. The clinical-pathological characteristics of the patients are summarised in Table I. The inclusion criteria were a pathological diagnosis of stage IV colorectal adenocarcinoma, an age ≥18 years and an Eastern Cooperative Oncology Group performance status <3. Patients treated prior to June 2009 were selected for CTX treatment on the basis of EGFR expression alone, as *KRAS* mutational status evaluation had not been made mandatory by the Italian Regulatory Authority at that time. All patients treated subsequent to June 2009 possessed tumours that were negative for *KRAS* mutations. This study was approved by the IRST Ethics Committee (Milan, Italy) and written informed consent was obtained from all patients.

Data were collected regarding patient characteristics, treatment and outcome. Treatment was continued until disease progression or toxicity occurred. The clinical response was assessed every eight weeks by a complete radiological examination, comprising a computed tomography (CT) or magnetic resonance imaging (MRI) scan, and it was also evaluated retrospectively according to the Response Evaluation Criteria in Solid Tumours guidelines (20). Objective tumour responses were classified into complete response, partial response, stable disease (SD) or progressive disease (PD). Patients with SD or PD were defined as non-responders. The objective response rate (ORR) was defined as the fraction of patients with complete or partial response confirmed ≥4 weeks after the initial response. Toxicity was evaluated according to the National Cancer Institute Common Terminology Criteria for Adverse Events v3.0 guidelines (21) for each patient receiving at least one dose of CTX-based chemotherapy.

### EGFR expression

The expression of EGFR was evaluated by immunohistochemistry performed on 5 μm-thick tissue sections obtained from paraffin-embedded tissue specimens, using the EGFR PharmaDx (Dako, Glostrup, Denmark) according to the manufacturer’s instructions.

### Gene mutation analyses

Formalin-fixed paraffin-embedded (FFPE) tumour blocks were reviewed for quality and tumour content. DNA was extracted from 5-μM FFPE sections of primary or metastatic lesions containing ≥50% tumour cells. Exon 2 of *KRAS*, exon 15 of *BRAF* and exons 9 and 20 of *PIK3CA* were analysed by pyrosequencing using the anti-EGFR moAb response kit for *KRAS*, *BRAF* and *PIK3CA* status (Diatech Pharmacogenetics, Ancona, Italy), respectively. The reactions were run on a PyroMark Q96 ID (Qiagen, Milan, Italy).

### EGFR methylation analysis

*EGFR* methylation status was evaluated by pyrosequencing analysis. In particular, three CpG islands were analysed using Hs_EGFR_02_PM PyroMark CpG assays (Qiagen). The analyses were performed on PyroMark Q96 ID (Qiagen).

### Statistical analysis

A two-sided Fisher’s exact test was used to evaluate the association between EGFR methylation, considered to be a dichotomous variable according to a cut-off of 10%, or the other gene mutations and ORR. The association between EGFR methylation, considered to be a continuous variable, and gene mutation was analysed using the Wilcoxon signed rank test.

The PFS rate was calculated from the first day of treatment to the date of the first observation of disease progression or, in the absence of progressive disease, the last follow-up or mortality. The OS rate was calculated from the first day of treatment to the date of mortality due to any cause or the date of the last follow-up. The PFS and OS rates and the 95% confidence interval (CI) were estimated using the Kaplan-Meier life-table method and the survival curves were compared using the log-rank test.

The impact of *EGFR* methylation on clinical outcome was evaluated in univariate analysis using a Cox regression model. All P-values were based on two-sided testing and statistical analyses were carried out using SAS statistical software (version 9.3; SAS Institute, Cary, NC, USA). P<0.05 was considered to indicate a statistically significant difference.

## Results

### EGFR expression

Analysis of EGFR expression was performed on 55 cases. In 34 patients, the analysis was only performed on the primary lesion, in 10 patients, only the metastatic lesion was analysed, and in 11 patients, the analysis was performed on each tumour lesion. Overall, 35 (64%) patients exhibited positivity for EGFR expression in at least one cell. In particular, EGFR expression was evident in 29 out of 45 (64%) primary tumours, and in 10 of the 21 (48%) metastatic lesions. Concordance between EGFR expression in primary and metastatic lesions was 27%, as only three cases out of 11 exhibited concordant expression. In all other cases, the primary tumour was positive for EGFR expression, whereas the metastatic lesion had lost EGFR expression.

### EGFR methylation

*EGFR* methylation analysis was performed on three CpG islands in all 64 mCRC patients. For 36 patients, the analysis was only performed on the primary lesion, for 14 patients, only the metastatic lesions were analysed, and for 14 patients, analysis was possible on each lesion. Where the analysis was performed on the two lesions, the results obtained on the metastatic lesion were taken into consideration for the overall statistical analysis.

Only two cases revealed no methylation in all three CpG islands. Assessment of the mean methylation in the three CpG islands revealed that 22 cases (34%) possessed a mean percentage of methylation of <10%, whereas 42 cases (66%) exhibited ≥10% methylation. In particular, 40 cases (63%) demonstrated a methylation rate between 10% and 50%, whereas two cases (3%) demonstrated an average percentage >50% in the primary or metastatic lesions. Separate analysis of the three CpG islands revealed that 16 samples (25%) exhibited a methylation level of <10% in all three islands, 48 cases (75%) exhibited a methylation level between 10 and 50% in at least one CpG island, and 13 cases (20%) possessed a methylation level >50% in one or more CpG islands. Of the 14 tumour samples for which the *EGFR* methylation analysis was performed on the primary and metastatic lesions, the results demonstrated heterogeneity between the two specimens. In particular, samples three and four clearly demonstrated an increased methylation level in the three CpG islands in the metastatic lesions, whereas sample six revealed a strong decrease at this site (Table II).

### KRAS, BRAF and PIK3CA mutation analyses

It was found that *KRAS* was mutated in 19 (30%) cases, comprising five G12V, three G12S, two G12D, one G12A, one G12C and seven G13D mutations. In three cases, a discordant result was obtained between the primary tumour and the metastatic lesion. In two of these cases, the primary tumour was wt and the metastatic lesion was mutated, whereas the remaining case demonstrated the opposite result, with mutation in the primary tumour and a wt metastatic lesion.

*BRAF* was mutated in 11 cases (17.2%). In all cases, the mutation was a V600E mutation on exon 15 of the gene. In four cases, a discordant result was observed between the primary tumour and metastatic lesion. In three of these cases, the mutation was evident in the metastatic lesion and not in the primary tumour, whereas in the remaining case, the mutation was evident in the primary tumour and not in the metastatic lesion. The *BRAF* mutation was associated with a shorter OS (6.9 months; 95% CI, 1.7–15.1) when compared with *BRAF* wt patients (10.0 months; 95% CI, 8.2–13.5) (P=0.09). However, no significant differences in PFS rate were identified between the two groups.

*PIK3CA* was mutated in seven cases (10.9%). In six cases the mutation was in exon 9 of the gene, consisting of one E545G, one E542K and four E545K mutations, whereas in the remaining case the mutation was in exon 20, with a H1047L mutation. In one case, the mutation was detected in the primary tumour and not in the metastatic lesion, whereas in the other cases, a concordant result was obtained between the two lesions. The *PIK3CA* mutation was associated with a shorter OS (7.0 months; 95% CI, 3.3–9.6) when comapred with *PIK3CA* wt patients (10.0 months; 95%, CI 8.3–13.7) (P=0.05). However, no significant differences in PFS rate were identified between the two groups.

### Correlation between *EGFR* methylation and other molecular alterations

In the primary tumour, no correlations were found between *KRAS*, *BRAF* or *PIK3CA* mutations and *EGFR* methylation, which were assessed in the three CpG islands separately or as mean value. Conversely, in the metastatic lesions, a correlation was found between the percentage of mean *EGFR* methylation and the presence of *KRAS* mutation, or the presence of a *BRAF*-wt gene. In particular, a mean *EGFR* methylation of 41% was observed in *KRAS*-mutated patients, compared to a methylation level of 9% in *KRAS*-wt patients (P=0.05). Conversely, a high *EGFR* methylation was observed in *BRAF*-wt patients compared with *BRAF*-mutated patients (18 vs. 3%, respectively; P=0.07) (Table III). No correlation was found between *EGFR* methylation and EGFR expression, in the primary and metastatic lesions.

### EGFR methylation in association with clinical response

The mean percentage of *EGFR* methylation in the three CpG islands demonstrated no correlation with the clinical response. Conversely, a higher median value of CpG island 2 methylation was observed in responders (22%) compared to non-responders (11%) (P=0.02), and the univariate analysis of objective response revealed a significant correlation (P=0.037).

**Figure 1:**
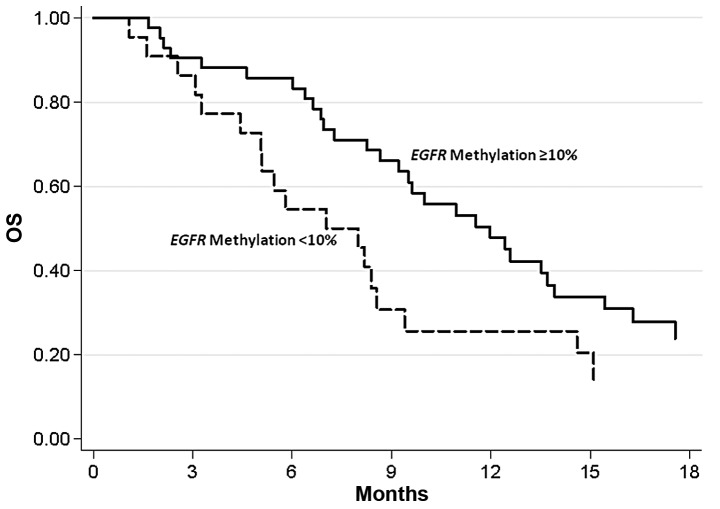
The association between OS rate and *EGFR* methylation. OS, overall survival.

In univariate analyses, with the EGFR methylation level being considered a continuous variable, the Cox regression model revealed that *EGFR* methylation was significantly correlated with OS rate (hazard ratio, 0.98; 95% CI, 0.96–1.00; P=0.019), but not with the PFS rate. In particular, the median OS rate of patients with a methylation rate <10% was 7.5 months (95% CI, 4.4–9.4 months) and the OS rate was 12.0 months (95% CI, 8.7–13.9 months) in patients with a methylation rate >10% (P=0.034) (Fig. 1).

## Discussion

Despite *KRAS* mutation being one of the selection criteria for the use of anti-EGFR therapy in mCRC patients, only a subgroup of *KRAS*-wt patients responded to the treatment. The analysis of other markers, including *NRAS*, *BRAF* and *PIK3CA*, have also allowed for the selection of further patients that may be likely to not respond to therapy (17,22). With a multiple marker selection, the probability of the selected patients responding to treatment may be significantly increased (4).

Although the target of moAb anti-EGFR drugs is the EGFR protein, several studies analysing the correlation between the expression or amplification of EGFR and clinical response have reported non-significant results (23–25). However, it is also likely that immunohistochemical evaluation of the EGFR protein expression may not be sufficiently accurate to detect the loss of EGFR protein in cancer tissue, thus compromising data analysis and interpretation (26). In addition, several biases may compromise the reproducibility of immunohistochemical analysis, including the different antibodies used and the varying reaction conditions that are used between different laboratories.

Gene methylation analysis may be an indirect approach towards analysing gene expression. It has been demonstrated that *EGFR* methylation is detectable in several types of solid tumour (27), but its association with EGFR immunohistochemical expression is unclear.

A previous study has demonstrated a significant correlation between the absence of *EGFR* methylation and the response to CTX-based chemotherapy in colorectal cancer patients. In particular, patients with a methylated gene were less responsive to therapy compared with patients without methylation, although no correlation between *EGFR* methylation and expression was identified (19). Conversely, in the present study, a significant correlation was found between the methylation of specific CpG islands and response to treatment, demonstrating that gene methylation was significantly correlated with ORR and OS rate, but not with PFS rate. The reasons for the discrepancy between the present study and the study by Scartozzi *et al* (19) may lie in the different CpG islands analysed and the different methodologies used. In particular, the present study analysed three CpG islands localised in the promoter region of *EGFR* (27), but these were upstream of the region analysed in the study by Scartozzi *et al*. In addition, pyrosequencing methodology was used in the present study, whereas the methylation-specific polymerase chain reaction approach was used in the aforementioned study.

To the best of our knowledge, the present study analysed the correlation between *EGFR* methylation and mutation in *KRAS*, *BRAF* and *PIK3CA,* which are genes involved in the resistance mechanisms to CTX, for the first time. No significant correlation was observed overall between *EGFR* methylation and the various gene mutations. However, it was only in metastatic lesions that gene methylation was found to be positively correlated with *KRAS* mutation and negatively correlated with *BRAF* mutation. In particular, a significantly increased percentage of methylation was observed in *KRAS*-mutated and *BRAF*-wt patients. Additionally, a high level of methylation was observed more frequently in *PIK3CA*-wt patients compared with *PIK3CA*-mutated patients, although this difference was not significant.

In the present study, the *BRAF* and *PIK3CA* mutations were correlated with a shorter OS rate, and the correlation identified between these mutations and the absence of *EGFR* methylation is consistent with the worse predictive value observed in patients with an *EGFR* methylation level <10%. The present study included patients with *KRAS-*wt and -mutated tumours that were treated with CTX prior to June 2009, and other patients selected for *KRAS*-wt tumours that were treated subsequent to June 2009. No significant correlations between the *KRAS* mutation status and the ORR, PFS rate and OS rate were identified, most likely due to the limited number of *KRAS-*mutated cases.

The correlations identified in metastatic lesions between *EGFR* methylation and *KRAS* mutation, may be due to the presence of constitutively activated signalling prompted by *KRAS* inducing cells to overcome the role of an EGFR hyperexpression. The inverse association identified with *BRAF* and *PIK3CA* mutations, by contrast, remains to be elucidated.

Similar to the study by Scartozzi *et al* (19), the present study did not identify any correlation between *EGFR* methylation and EGFR expression, despite the varying results obtained in terms of methylation.

In conclusion, the present study demonstrated correlations between *EGFR* methylation and an improved response and OS rate in patients treated with CTX-based chemotherapy. The presence of *EGFR* methylation was found to be inversely correlated with *BRAF* and *PIK3CA* mutations, indicating that the prognostic value of gene methylation requires additional verification in future studies.

## Figures and Tables

**Table I:** Baseline patient characteristics.

Characteristic	Value
Number of patients, n	64
Age, years	
Median	61
Range	34–79
Gender, n (%)	
Male	37 (57.8)
Female	27 (42.2)
Performance status, n (%)	
0	38 (59.4)
1–2	26 (40.6)
Primary tumour site, n (%)	
Colon	51 (79.7)
Rectum	13 (20.3)
Treatment regimen, n (%)	
CTX+irinotecan/FOLFIRI	57 (89.1)
CTX+FOLFOX4	6 (9.4)
CTX alone	1 (1.6)
Previous chemotherapy, n (%)	
Irinotecan-based	59 (92.2)
Fluoropyrimidine-based	64 (100.0)
Oxaliplatin-based	52 (81.3)
Bevacizumab-based	23 (35.9)
Previous cancer treatments for advanced disease, n (%)	
1	14 (21.9)
2	26 (40.6)
3	15 (23.4)
>3	9 (14.1)
Cutaneous toxicity, n (%)	
0	16 (25.0)
1	19 (29.7)
2–3	29 (45.3)

FOLFIRI, folinic acid, 5-fluorouracil and irinotecan hydrochloride; FOLFOX4, folinic acid, 5-fluorouracil, and oxaliplatin.

**Table II:** Methylation of three *EGFR* CpG islands of in the primary tumor and matched metastatic lesions.

	Methylation of primary tumor tissue			Methylation of metastatic lesion		
		
Sample	CpG1, %	CpG2, %	CpG3, %	CpG1, %	CpG2, %	CpG3, %
1	5	7	14	0	3	0
2	7	55	15	18	32	74
3	0	9	0	4	82	84
4	7	9	0	48	49	54
5	5	0	6	10	0	0
6	65	61	63	0	4	0
7	9	29	35	14	31	35
8	8	16	17	16	24	17
9	0	0	25	0	9	3
10	2	27	36	0	5	4
11	1	8	13	25	0	0
12	27	21	23	2	0	68
13	65	3	0	2	12	14
14	17	21	32	40	61	33

**Table III:** Correlation between *EGFR* methylation and *KRAS*, *BRAF* and *PIK3CA* mutations.

		CpG1 methylation		CpG2 methylation		CpG3 methylation		Mean methylation	
					
Mutation status	Number of patients	Median value (range)	P-value	Median value (range)	P-value	Median value (range)	P-value	Median value (range)	P-value
Primary tumor (n=54)									
*KRAS* wt	39	8.0 (0–65)		13.0 (0–55)		14.0 (0–61)		17.0 (0–42)	
*KRAS* mut	15	7.0 (0–65)	0.516	17.0 (4–61)	0.077	15.0 (0–63)	0.582	13.0 (3–63)	0.578
*BRAF* wt	46	8.5 (0–65)		15.0 (0–61)		16.5 (0–63)		15.5 (0–63)	
*BRAF* mut	8	6.0 (2–65)	0.773	3.0 (0–37)	0.217	6.0 (0–61)	0.335	17.0 (2–42)	0.529
*PIK3CA* wt	47	9.0 (0–65)		15.0 (0–56)		14.0 (0–61)		17.0 (0–54)	
*PIK3CA* mut	7	1.5 (0–65)	0.186	10.5 (0–61)	0.429	13.0 (0–63)	0.791	5.5 (0–63)	0.111
Metastatic lesions (n=29)									
*KRAS* wt	18	4.0 (0–34)		9.0 (0–25)		7.0 (0–68)		9.0 (0–26)	
*KRAS* mut	11	18.0 (0–48)	0.034	32.0 (0–82)	0.072	29.0 (0–84)	0.221	41.0 (1–57)	0.057
*BRAF* wt	23	12.5 (0–48)		13.5 (0–82)		15.5 (0–84)		18.0 (0–57)	
*BRAF* mut	6	5.0 (0–10)	0.365	0.0 (0–5)	0.051	4.0 (0–4)	0.114	3.0 (3–3)	0.077
*PIK3CA* wt	26	12.5 (0–48)		11.5 (0–62)		12.5 (0–74)		14.0 (0–50)	
*PIK3CA* mut	3	4.0 (0–4)	0.178	10.0 (4–82)	0.740	7.0 (0–84)	1.000	7.0 (1–57)	0.803

mut, mutation; wt, wild-type.
